# Screening Accuracy for Developmental Dysplasia of the Hip by Child Health Nurses

**DOI:** 10.3390/children11080915

**Published:** 2024-07-29

**Authors:** Larissa Smart, William Cundy, Nicole Williams, Abigail Arnold, Jessie Childs, Lemuel Pelentsov, Adrian Esterman

**Affiliations:** 1 School of Nursing, University of Tasmania, Locked Bag 3513, Burnie, TAS 7320, Australia; 2 Clinical and Health Sciences, University of South Australia, Adelaide, SA 5000, Australia; 3 Women’s and Children’s Health Network, SA Health, Adelaide, SA 5000, Australia; 4 Allied Health and Human Performance, University of South Australia, Adelaide, SA 5000, Australiaadrian.esterman@unisa.edu.au (A.E.)

**Keywords:** child health, developmental dysplasia of the hip, accuracy, infant, child

## Abstract

Background: Developmental Dysplasia of the Hip (DDH) describes a spectrum of abnormalities that impact the normal function of the joint. These abnormalities are detectable during infancy using physical assessment, universal ultrasound, or a combination of both. In Australia, child health nurses predominantly screen for this disease using physical assessment. The aim of this study was to determine if child health nurses can accurately screen for DDH using physical assessment. Methods: This Australian study estimated the accuracy and utility of child health nurses using physical assessment to screen for DDH by comparing it to a handheld ultrasound device. Results: This study (N = 44) concluded that using a point prevalence study design, child health nurses in Australia are unable to accurately assess for DDH in infants using physical assessment; overall, clinicians had 50.5% sensitivity (95% CI, 15.7 to 84.3), a specificity of 75.0% (95% CI, 57.8 to 87.9), and 71.3% accuracy (95% CI, 55.6 to 83.9). Conclusion: This study suggests that child health nurses cannot screen for DDH using physical assessment. Understanding education, experience and what assessments are used suggests that the utility of nurses performing DDH screening requires further investigation.

## 1. Introduction

**Figure 1:**
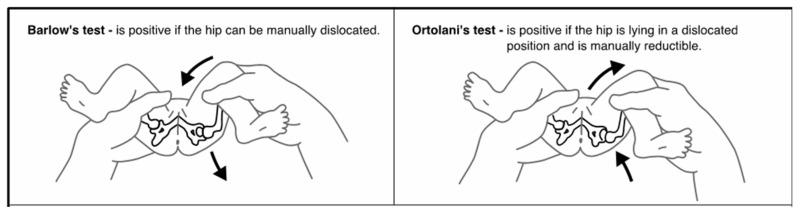
Barlow and Ortolani Manoeuvres [5].

Developmental Dysplasia of the Hip (DDH) describes a spectrum of abnormalities in which the femoral head and acetabulum are not in alignment, grow abnormally, or exhibit a combination of both [1]. These abnormalities can occur in utero, during the perinatal period, or during infancy and early childhood as a result of both genetic and environmental factors [2,3]. This etiology results in an unstable or dislocatable hip, which, without early diagnosis and treatment, can have lifelong implications for the child [1]. Universally, the most accepted screening method for DDH is the use of physical assessment [2,4]. The aim of this screening program is to identify any abnormalities that indicate DDH as early as possible to begin treatment. The earlier treatment begins, often the less invasive and shorter the duration. Infants who do not have their DDH detected and treated are at risk of long-term morbidity, pain, and disability [5]. A recent review of the literature identified 18 different assessments that were utilized by clinicians as part of this DDH screening, the most common of which are the Barlow Manoeuvre, Ortolani Manoeuvre, and assessment of the infant’s legs [1]. The Barlow Manoeuvre, first described in 1962, involves adducting the infant’s hip using forward pressure to determine if the hip can be manually dislocated [5,6]. The Ortolani Manoeuvre, first described in 1936, involves using adduction and then abduction to manually reduce an already dislocated hip [7] (Figure 1). Finally, assessment of an infant’s legs includes visual inspection for asymmetrical skin folds, testing for the Galeazzi sign and assessment of leg length [1]. Despite these maneuvers being the most common, they have been shown to have low sensitivity and poor positive predictive value [8,9].

Targeted and universal ultrasound imaging of the hip during screening for DDH is used internationally; however, there is yet to be widespread agreement on the best approach to ultrasound screening [2]. Despite this disparity, universal ultrasound screening has been proven to be a sensitive and effective screening tool [10] and is currently used for all infants in many European countries [11] and thus could be considered the gold standard. In Australia, screening for DDH using physical assessment is recommended shortly after birth and routinely during the first 12 months of life [12,13,14,15,16]. Whilst any clinician trained in physical assessment of the hips can screen for this condition, the screening is prioritized and most often performed by child health nurses at universal well-child health checks [1,13,15,17]. Infants with any abnormal findings are referred to general practitioners or orthopedic surgeons for further investigation, diagnosis, and treatment [18]. Treatment for DDH is dependent on the type and cause of the DDH, the age and development of the infant, and the degree of hip displacement [5]. The purpose of treatment is, however, the same: to use the least invasive method to retain the femoral head in the acetabulum while the ligaments and bone return to normal [19].

Despite child health nurses being the predominant clinician to screen infants for this condition in Australia, there is currently no published evidence as to the screening accuracy of child health nurses using physical assessment to screen for DDH. The aims of this study were, therefore, to determine whether child health nurses can accurately screen for DDH in infants using physical assessments and the utility of nurses performing these assessments as part of the universal child health program.

## 2. Materials and Methods

This study received ethics approval from the University of South Australia Human Research Ethics Committee (Study ID 204517) and the South Australia Health Human Research Ethics Committee and associated site-specific governance (Study ID 00038). All participants, including clinicians and parents, provided informed written consent to participate in the study prior to any involvement.

The report for this study utilized the Standards for Reporting Diagnostic Accuracy (STARD) checklist [20,21].

### 2.1. Study Design

This was an Australian study estimating accuracy of physical screening assessments for DDH. Child health nurses included in the study were identified by the area’s nurse educator prior to the commencement of the study. An information session was held to introduce the child health nurses to the study protocol and clinic processes.

The parents of infants in the study were approached by researchers in the child health clinic. All infants that were included in the study had their normal child health appointment, which included a physical assessment of the hip to screen for DDH. During the appointment, the child health nurse indicated on a data card if the child’s physical assessment was normal or abnormal and which physical assessments were used. Directly after the physical assessment, a clinician who was experienced in hip ultrasound took bilateral images of the hip using a portable handheld ultrasound device. The ultrasound images were then analyzed to determine hip position, acetabular morphology, femoral head coverage, and the alpha angle, which were measured to classify the hip according to Graf’s method [22]. The findings of this ultrasound were either identified as normal or abnormal on a second data card, and the alpha angles were recorded. Parents were informed of the combined findings of the assessments, and any child found to have any abnormal finding, regardless of test, was referred to the Women’s and Children’s Hospital hip clinic via the area’s normal referral pathway.

### 2.2. Participants

There were two target populations for the study: (1) child health nurses who were assessing infants in the pre-identified clinics and (2) infants attending the child health clinic. Parents were approached by the research team in the child health clinic prior to or during their appointment. Any parents whose infant met the eligibility criteria and provided consent were included in the study. All participants consented and received the physical and ultrasound assessment on the same day. Participants were recruited at seven child health clinics in metropolitan and regional centers in one state of Australia during October 2023.

### 2.3. Inclusion Criteria

All child health nurses included in the study were required to screen infants for DDH who had consented to participate in the project. Infants included in the study had to be under the age of 12 months and were not yet independently walking. The infants must not have been previously diagnosed with or treated for DDH. Finally, the infants had to attend the child health clinic for a universal well-child assessment.

### 2.4. Exclusion Criteria

Infants with parents who were unable to provide consent in English were excluded from the study.

### 2.5. Test Methods

The child health nurse’s physical assessment for detecting DDH was performed on all infants as part of their universal well-child assessment. The child health nurses were asked to do the physical assessment as per their normal practice. A previous review of the literature identified that the most common components of a child health nurse’s physical assessment include the Barlow Manoeuvre, the Ortolani Manoeuvre, asymmetrical skin folds, leg length discrepancy, limitation of abduction, the Galeazzi sign, the range of movement, and infant history [1]. These eight components were identified on the data collection card, as well as an ‘other’ item for child health nurses to identify how they had conducted the physical assessment. The child health nurse indicated their physical assessment findings on the first data card and placed it into a participant envelope.

Immediately after the physical assessment, the research team was invited into the clinic room to perform the hip ultrasound. All hip ultrasounds were performed by a pediatric orthopedic surgeon trained in the Graf technique.

The infant was held in the right lateral position and then the left lateral position to allow for bilateral hip images and identification of anatomical landmarks. Prior to each image, a small amount of water-soluble ultrasound gel was applied to each hip. Each hip took approximately 30 s for the surgeon to assess. An image was saved onto an iPad and then reviewed by the surgeon according to the published Graf criteria, using a digital ruler and goniometer to measure the alpha angle [22]. The surgeon indicated their ultrasound findings (normal hip development vs. hip clinic referral required) on a second data card and placed it into the participant’s envelope. Both clinicians were blinded to the other clinician’s assessment until after the ultrasound result had been provided to the research team. Scripted responses were then stated by the surgeon to indicate the ultrasound findings to the child health nurse, who would then communicate the combined assessment findings to the parents and make any required referrals to the hip clinic.

Additionally, demographic information for each participant was collected. Information collected about infants included date of birth, gender, single or multiple pregnancy, and if the infant was a first child. Child health nurses provided gender, age range, current clinical position, highest level of education, and years of experience assessing for DDH.

### 2.6. Statistical Analysis

Descriptive statistics were used to present demographic information on the two participant population groups. Frequency distribution of participants’ responses to the demographic questionnaire was determined to summarize the collected data.

Statistical analysis of the assessment outcome (positive or negative abnormal findings) was conducted to determine sensitivity, specificity, positive and negative predictive values, positive and negative likelihood ratios, and accuracy using SPSS (IBM SPSS Statistics for Windows, Version 28.0., IBM Corp.: Armonk, NY, USA) and MedCalc (MedCalc Statistical Software version 2: MedCalc Software bv, Ostend, Belgium) software packages. Finally, the frequency distribution and accuracy of assessments with abnormal findings were determined to provide relevance to current clinicians in practice. The prevalence data used in this study to analyze and report the outcomes were 15%. This prevalence is based on the prevalence of hip instability, hip immaturity and hip abnormalities that require detection and monitoring [23]. All data analyses were completed by the first and last authors.

## 3. Results

### 3.1. Participants

In total, 15 child health nurses consented to be part of the study. The majority of nurses were aged 51+ years. Most indicated that they had 1–5 years of experience assessing for DDH and had either a Bachelor’s Degree or Graduate Certificate level of education (Table 1).

**Figure 2:**
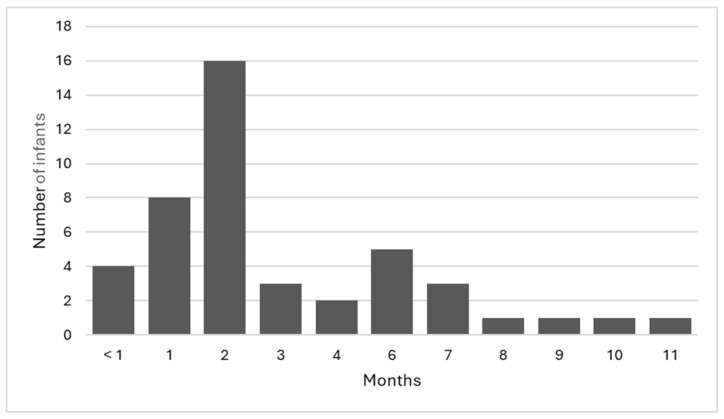
Age of infants.

There were 44 infants with parental consent to participate in the study. Of the 44 infants, 65.9% (n = 29) were female, 90.9% (n = 40) were singleton pregnancies, with the remaining 9.1% (n = 4) being twin pregnancies, and 72.7% (n = 32) of the infants were the first child for the mother. The infants included in the study were aged between <1 month and 11 months of age (Figure 2), with a mean age of 3.1 months. Overall, 36.4% (n = 16) of the infants were in the 2-month age bracket; this age range coincides with the ‘8-week check’ that is part of the geographical area’s recommended universal well-child assessment.

### 3.2. Test Results

Overall, all clinicians had 50.5% sensitivity (95% CI, 15.7 to 84.3), a specificity of 75.0% (95% CI, 57.8 to 87.9), and 71.3% accuracy (95% CI, 55.6 to 83.9 when using physical assessment to detect abnormal findings indicating DDH compared with the gold standard of ultrasound. Screening accuracy was also assessed when clinicians were stratified by five variables, and sensitivity ranged from 0 to 66.7% and specificity from 70.6 to 83.3% for all five variables. There was little difference when clinician years of experience were used as a variable. However, when the level of educational attainment was used to stratify clinicians, those with postgraduate qualifications had higher sensitivity (66.7%), although overall, there was no significant change in accuracy. Accuracy ranged from 67.5 to 74.6%; clinicians with 11+ years of experience were the most accurate, and clinicians with <11 years of experience were the least accurate (Table 2).

An overall positive predictive value (PPV) of 26.1% indicates that a positive screening assessment is a poor indication that DDH is actually present and that there were many false positive findings. However, the overall negative predictive value (NPV) of 89.5% indicates that a negative screening assessment is likely a true negative and that DDH is not present. The positive likelihood ratio (PLR) of 2.0 indicates that a positive physical assessment only marginally increases the likelihood that the infant actually has DDH. The overall negative predictive ratio (NLR) of 0.67 shows that a negative physical assessment only marginally increases the likelihood that the infant does not have DDH.

**Figure 3:**
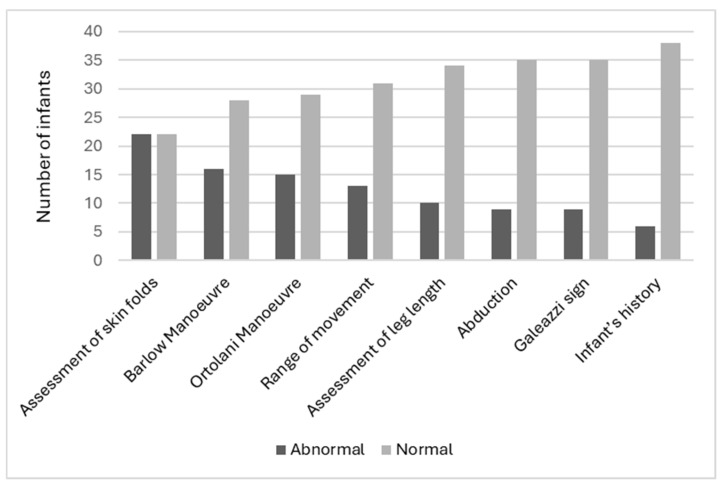
Abnormal test findings.

Clinicians indicated the most abnormal assessment finding was the assessment of skin folds (50.0%, n = 22), followed by the Barlow Manoeuvre (36.4%, n = 16) and the Ortolani Manoeuvre (34.1%, n = 15). The least abnormal assessment was the infant’s history (13.6%, n = 6) (Figure 3). It should be noted that not all abnormal skin fold findings were considered overall abnormal findings by the clinician for that infant’s assessment. This was clarified by the research team at the time of data collection, and clinicians determined that abnormal gluteal folds lead to a high suspicion of DDH; however, abnormal thigh folds and lower limb folds did not. It should be noted that no clinicians utilized the ‘other’ section on the data card. All child health nurses who participated expressed interest to the research team about the use of ultrasound screening and what it may mean to their scope of practice. Finally, the true positive prevalence of abnormal hip findings was 18.2% (n = 8).

## 4. Discussion

The findings of this study suggest that using physical assessment for screening infants for DDH is not accurate when performed by child health nurses. As DDH is a spectrum of abnormalities, sensitivity and the ability to detect infants with signs of DDH may be more important than specificity. The low overall sensitivity (50.5%) shows that almost half of the infants with abnormal hip findings were not detected using physical assessment alone. Clinicians with educational attainment beyond a Bachelor’s Degree had higher sensitivity than clinicians without, although overall, there was no significant change in accuracy. Several physical assessments were consistently found to be abnormal during screening, with the most common being abnormal skin folds, the Barlow Manoeuvre, and the Ortolani Manoeuvre.

The accuracy of DDH screening programs is universally disputed in the literature [8]. Recent international research has concluded that when clinicians such as orthopedic surgeons, general practitioners, pediatric consultants, and other treating hip specialists screen for DDH, there is low sensitivity and PPV [8]. This study has found that this is true in the Australian context and that child health nurses, similarly to their international screening counterparts, cannot accurately detect signs of DDH. The Population-Based Screening Framework [24] outlines seven criteria that must be met in order for a screening test to be acceptable, including high sensitivity, high specificity, high PPV, and high NPV. The results of this research demonstrate that DDH screening in Australia currently does not meet these criteria. The slight increase in sensitivity of clinicians with postgraduate qualifications indicates that training and education when assessing for DDH is essential for screening accuracy. To be clear, it is the physical assessment methods used by Australian child health nurses that are inaccurate, and this would also be true when these assessments were used by other health professionals.

A 2022 review of the literature [1] found that accurate screening for DDH required extensive understanding and skill related to DDH anatomy, risk factors, and specific physical screening assessments. The high percentage of infants with abnormal skin folds (50.0%, n = 22) in this study has been replicated in the literature. Abnormal or asymmetrical skin folds are commonly seen in both the medial thigh and gluteal areas [25]. This abnormal finding is the most common reason for referral during the screening assessment; however, it is a poor indicator of DDH [25]. The Barlow Manoeuvre was clinically abnormal in 36.4% (n = 16) of screening assessments. Diagnostically, the Barlow Manoeuvre has been found to have a PPV of only 22% [26]. While the Ortolani Manoeuvre was abnormal in 34.1% (n = 15) of infants, when combined with the Barlow Manoeuvre, it has been found to be clinically normal in over 60% of infants that require treatment for DDH [8]. This means that the three most common abnormal findings in this study have no to poor evidence that they are sensitive or accurate in detecting signs of DDH.

Universal ultrasound screening for DDH using the Graf technique has been found to be an accurate, sensitive tool to identify infants with signs of DDH. A US study found that universal ultrasound screening detected late-diagnosed DDH in all cases [10]. Additional studies investigating the use of artificial intelligence and ultrasound to detect DDH found the use of an automated system to measure and define abnormalities was accurate, fast, and robust, with a sensitivity of 90.56% and a specificity of 100% [27]. At the same time, a recent systematic review found that six European nations perform universal ultrasound screening to detect DDH [28].

The World Health Organization defines screening as a process for identifying individuals in the population who are at high risk of a health condition to enable early treatment and better health outcomes for the individual and overall population [29]. A useful screening program should be highly sensitive and specific to accurately identify individuals with the condition [29]. A screening program for DDH that is not accurate has long-term implications for infants, families, and healthcare systems. When DDH is left undetected and, therefore, untreated, it may lead to early-onset arthritis, pain, and lifelong disability [30]. When treatment is provided early, it is typically less invasive, has a shorter duration, and is curative [31]. The current physical assessment methods for screening for DDH are not accurate, and therefore, further research into a screening program that is highly accurate is required.

### Limitations

This research had several limitations. First, the sample sizes of both participant groups were relatively small. Secondly, the data were collected in one geographical area. Thirdly, there was no inter and intra-screener reliability. Fourth, there was no follow-up on infants referred for further assessment and diagnosis or long-term follow-up of infants with a negative screening result.

The small sample size in this study can be attributed to the combined availability of child health clinics and the pediatric orthopedic surgeon. Despite continued attempts to facilitate combined availability, it was not achievable. 

Our findings indicate that further studies with larger sample sizes are required. A further study could confirm the findings of this study and address the limitations identified.

## 5. Conclusions

The findings from this study suggest that screening accuracy for DDH using physical assessment is of limited value. A larger study would confirm this. Understanding education, experience, and what assessments are used suggests that the utility of nurses performing DDH screening requires further investigation.

It appears that the techniques currently used are not accurate for screening in practice, and further study is needed to understand the education and training clinicians receive prior to screening for DDH. Furthermore, universal ultrasound screening and the use of artificial intelligence may increase the utility of a continuing DDH screening program that meets the criteria of a screening program. Innovative changes to practice with validated, evidence-based outcomes that are cost-effective and highly sensitive would ensure that Australian infants are among the first in the world to detect, diagnose and treat DDH as early as possible.

## Figures and Tables

**Table 1:** Child health nurse demographics.

Characteristics		N	%
Age	21–30 years	2	13.3
	31–40 years	3	20.0
	41–50 years	3	20.0
	51+ years	7	46.7
	Total	15	100.0
Position	Registered Nurse	2	13.3
	Child Health Nurse	6	40.0
	Midwife and Registered Nurse	1	6.7
	Midwife and Child Health Nurse	6	40.0
	Total	15	100.0
Education	Bachelor’s Degree	6	40.0
	Graduate Certificate	6	40.0
	Graduate Diploma	3	20.0
	Total	15	100.0
Experience	1–5 years	7	46.7
	6–10 years	3	20.0
	11–15 years	2	13.3
	16–20 years	2	13.3
	21+ years	1	6.7
	Total	15	100.0

**Table 2:** Test by clinician information.

Variable	N *	Sensitivity (%)	Specificity (%)	PLR	NLR	PPV (%)	NPV (%)	Accuracy (%)
All clinicians	44	50.5	75.0	2.0	0.67	26.1	89.5	71.3
95% confidence interval		15.7–84.3	57.8–87.9	0.8–4.9	0.3–1.4	12.6–46.3	80.6–94.6	55.6–83.9
Clinicians with <11 years of experience	23	50.0	70.6	1.7	0.71	23.1	88.9	67.5
95% confidence interval		11.8–88.1	44.0–89.7	0.6–5.0	0.3–1.7	9.2–47.1	77.3–95.0	45.0–85.3
Clinicians with 11+ years of experience	21	50.0	78.9	2.4	0.6	29.5	89.9	74.6
95% confidence interval		1.2–98.7	54.4–93.9	0.5–12.2	0.2–2.6	7.5–68.3	68.7–97.3	51.4–90.8
Clinicians with Bachelor’s Degree **	14	0.0	83.3	0.0	1.2	0.0	82.5	70.8
95% confidence interval		0.0–84.2	51.6–97.9	–	0.9–1.5	0.0–84.2	78.6–85.9	41.3–91.3
Clinicians with postgraduate qualification	30	66.7	70.8	2.3	0.5	28.7	92.3	70.2
95% confidence interval		22.3–95.7	48.9–87.4	1.0–5.3	0.1–1.5	14.8–48.4	79.0–97.5	50.8–85.4

Notes: * N = number of assessments completed by each variable group. ** No clinicians with a Bachelor’s Degree detected any abnormal findings during their assessment. This explains the 0.0 values and missing 95% confidence interval for PLR. Abbreviations: PLR—positive likelihood ratio, NLR—negative likelihood ratio, PPV—positive predictive value, NPV—negative predictive value.

## Data Availability

The raw data supporting the conclusions of this article will be made available by the authors on request.

## References

[1] Smart L., Pelentsov L., Childs J., Williams N., Esterman A. (2024). Nurses assessment of development hip dysplasia: A scoping review. J. Child Health Care.

[2] Lehmann H.P., Hinton R., Morello P., Santoli J. (2000). Developmental dysplasia of the hip practice guideline: Technical report. Pediatrics.

[3] Shipman S.A. (2006). Screening for Developmental Dysplasia of the Hip: A Systematic Literature Review for the US Preventive Services Task Force. Pediatrics.

[4] Marriott E., Twomey S., Lee M., Williams N. (2021). Variability in Australian screening guidelines for developmental dysplasia of the hip. J. Paediatr. Child Health.

[5] Williams N. (2018). Improving early detection of developmental dysplasia of the hip through general practitioner assessment and surveillance. Aust. J. Gen. Pract..

[6] Barlow T. (1962). Early diagnosis and treatment of congenital dislocation of the hip. J. Bone Jt. Surg. Br. Vol..

[7] Ortolani M. (1937). Un segno poco noto e sua importanza per la diagnosi precose di prelussazione congenita dell’anca. Pediatrica.

[8] Paton R.W. (2017). Screening in developmental dysplasia of the hip (DDH). Surgeon.

[9] Tan S.H.S., Lim J.X.Y., Lim A.K.S., Hui J.H.P. (2023). Risk factors for a false negative Ortolani and Barlow examination in developmental dysplasia of the hip. Orthop. Traumatol. Surg. Res..

[10] Gyurkovits Z., Sohár G., Baricsa A., Németh G., Orvos H., Dubs B. (2021). Early detection of developmental dysplasia of hip by ultrasound. Hip Int..

[11] Kilsdonk I., Witbreuk M., Van Der Woude H.-J. (2021). Ultrasound of the neonatal hip as a screening tool for DDH: How to screen and differences in screening programs between European countries. J. Ultrason..

[12] Department of Health (2017). My Health, Learning and Development.

[13] NSW Ministry of Health (2021). My Personal Health Record.

[14] NT Health (2018). NT Child Health Record.

[15] Queensland Health (2018). Personal Health Record.

[16] WA Health (2019). Purple Book.

[17] ACT Health (2018). My ACT Personal Health Record.

[18] (2017). Neonatal Hip Screening and Management of Developmental Dysplasia of the Hip. https://www.sahealth.sa.gov.au/wps/wcm/connect/59837e804ee50abc982e9dd150ce4f37/Neonatal+Hip+Screening+and+Manage-ment+of+Developmental+Dysplasia+of+the+Hip_PPG_v2.pdf?MOD=AJPERES&CACHEID=ROOTWORKSPACE-59837e804ee50abc982e9dd150ce4f37-ocQW.IT.

[19] International Hip Dysplasia Institute (2024). Understanding Hip Dysplasia.

[20] Bossuyt P.M., Reitsma J.B., Bruns D.E., Gatsonis C.A., Glasziou P.P., Irwig L.M., Lijmer J.G., Moher D., Rennie D., de Vet H.C.W. (2003). Towards complete and accurate reporting of studies of diagnostic accuracy: The STARD initiative. BMJ.

[21] Šimundić A.-M. (2009). Measures of diagnostic accuracy: Basic definitions. EJIFCC.

[22] Graf R. (2006). Hip Sonography: Diagnosis and Management of Infant Hip Dysplasia.

[23] Shaw B.A., Segal L.S., Otsuka N.Y., Schwend R.M., Ganley T.J., Herman M.J., Hyman J.E., Shaw B.A., Smith B.G., Section on Orthopaedics (2016). Evaluation and referral for developmental dysplasia of the hip in infants. Pediatrics.

[24] Australian Government (2020). Population Based Screening Framework.

[25] Kang M.S., Han G.W., Kam M., Park S.-S. (2019). Clinical significance of asymmetric skin folds in the medial thigh for the infantile screening of developmental dysplasia of the hip. Pediatr. Neonatol..

[26] Patel H. (2001). Preventive health care, 2001 update: Screening and management of developmental dysplasia of the hip in newborns. Can. Med. Assoc. J..

[27] Huang B., Xia B., Qian J., Zhou X., Zhou X., Liu S., Chang A., Yan Z., Tang Z., Xu N. (2023). Artificial Intelligence-Assisted Ultrasound Diagnosis on Infant Developmental Dysplasia of the Hip Under Constrained Computational Resources. J. Ultrasound Med..

[28] Krysta W., Dudek P., Pulik Ł., Łęgosz P. (2024). Screening of Developmental Dysplasia of the Hip in Europe: A Systematic Review. Children.

[29] World Health Organization (2020). Screening Programmes: A Short Guide Increase Effectiveness, Maximize Benefits and Minimize Harm.

[30] Nicholson A., Dunne K., Taaffe S., Sheikh Y., Murphy J. (2023). Developmental dysplasia of the hip in infants and children. BMJ.

[31] Canavese F., Castañeda P., Hui J., Li L., Li Y., Roposch A. (2020). Developmental dysplasia of the hip: Promoting global exchanges to enable understanding the disease and improve patient care. Orthop. Traumatol. Surg. Res..

